# Physiochemical Characterization and Antioxidant Potential of Sorghum and Cork Oak as Valuable Additives to Traditional *Trida* Pasta

**DOI:** 10.3390/foods14162832

**Published:** 2025-08-15

**Authors:** Rima Sabouni, Louiza Himed, Belkis Akachat, Agnieszka Wójtowicz, Kamila Kasprzak-Drozd, Hacène Namoune, Salah Merniz, Maria D’Elia, Luca Rastrelli, Anna Oniszczuk

**Affiliations:** 1 Laboratoire de Nutrition et Technologie Alimentaire, Institut de la Nutrition, de l’Alimentation et des Technologies Agro-Alimentaires, Université des Frères Mentouri-Constantine 1, Route de Ain El-Bey, Constantine 25000, Algeria; ryma.sabouni@umc.edu.dz (R.S.); namounh54@yahoo.fr (H.N.); 2 Laboratory of Biotechnology and Food Quality (BIOQUAL), Department of Food Biotechnology, Institute of Nutrition, Food and Agro-Food Technologies (INATAA), Université Frères Mentouri-Constantine 1, Route de Ain El-Bey, Constantine 25000, Algeria; louiza.himed@umc.edu.dz (L.H.); belkis.akachat@doc.umc.edu.dz (B.A.); 3 Department of Food Process Engineering, University of Life Sciences in Lublin, Głęboka 31, 20-612 Lublin, Poland; 4 Department of Inorganic Chemistry, Medical University of Lublin, Chodźki 4a, 20-093 Lublin, Poland; kamilakasprzakdrozd@umlub.pl (K.K.-D.); anna.oniszczuk@umlub.pl (A.O.); 5 Institute of Industrial Hygiene and Safety, University Batna 2, Batna 05078, Algeria; merniz@univ-batna2.dz; 6 Department of Pharmacy, University of Salerno, Via Giovanni Paolo II, 132, Fisciano, 84084 Salerno, Italy; mdelia@unisa.it; 7 National Biodiversity Future Center (NBFC), 90133 Palermo, Italy; 8 Dipartimento di Scienze della Terra e del Mare, University of Palermo, 90133 Palermo, Italy

**Keywords:** *Trida* pasta, sorghum flour, cork oak flour, nutritional enhancement, antioxidant capacity, cooking behavior, texture profile, microstructure

## Abstract

This study aimed to valorize underutilized local ingredients by developing nutritionally enhanced pasta products enriched with sorghum and cork oak flours. The resulting pasta samples were characterized by their chemical composition, color attributes, functional properties, texture, microstructure, and antioxidant capacity. Semolina-based pasta showed higher protein content, while cork oak flour contributed significantly to lipid content, and sorghum flour was notably rich in fiber and minerals. Colorimetric analysis quantified visible differences in appearance, depending on the type of flour used. Functional assessment showed comparable water absorption indices across all samples; however, sorghum-enriched pasta exhibited significantly higher water solubility. Textural analysis indicated that sorghum reduced pasta adhesiveness and cohesiveness, whereas cork oak flour increased hardness, gumminess, and adhesiveness—likely due to its high fiber content, contributing to a stickier mouthfeel. Microstructural observations confirmed a denser and more compact matrix in pasta formulated with cork oak flour. Antioxidant analysis revealed that cork oak flour imparted the highest antioxidant potential, followed by sorghum and semolina. HPLC/ESI-TOF-MS profiling demonstrated a rich and diverse polyphenolic composition in the enriched samples. These formulations not only enhance the functional and nutritional profile of traditional pasta but also align with the increasing consumer demand for low-carbohydrate, fiber-rich foods.

## 1. Introduction

Dry pasta is a staple food consumed globally and holds particular importance in Mediterranean diets, where it is valued for its texture and versatility. Traditionally, pasta is made from durum wheat semolina, which remains the main ingredient in conventional recipes [1]. The production of pasta typically involves a series of unit operations, including hydration, mixing, extrusion or lamination, and drying [2]. In Algeria, durum wheat is a fundamental part of the national diet and is used to produce a variety of foods such as industrial pasta, couscous, flatbreads, and other traditional products. Surveys have documented the wide range of pasta types consumed across the country, especially in the eastern regions [3]. Among these, *Trida* is a notable traditional pasta, resembling Italian tagliatelle, and is widely enjoyed both in homemade preparations and, increasingly, by commercial producers. Recently, there has been growing interest in enhancing the nutritional value of pasta by incorporating bioactive ingredients beyond durum wheat [4]. Legume flours, barley, dietary fibers, proteins, and omega-3 fatty acids have all been explored as functional additions to improve pasta’s nutritional profile [5]. These modifications not only increase the nutritional content but also affect the technological and sensory properties of the final product. At the same time, there is increasing consumer demand for low-carbohydrate, high-fiber foods with a reduced glycemic impact, driven by health concerns such as metabolic syndrome, diabetes, and weight management. Historically, during periods of food scarcity such as World War II, Algerian and Moroccan communities turned to alternative ingredients like acorn flour for making couscous and flatbreads [6]. Acorn flour from cork oak (*Quercus suber* L.) is particularly rich in unsaturated fatty acids, especially oleic acid (>63%), followed by palmitic and linoleic acids at similar concentrations (12–20%), as well as dietary fiber, minerals, and antioxidants [7,8]. It provides slow-digestible carbohydrates and has a low glycemic index, making it an attractive ingredient for health-conscious consumers [8]. Sorghum (*Sorghum bicolor*) exhibits a highly favorable nutritional profile, with protein content ranging from 9.0% to 12.5%, fiber content between 1.17% and 7.73%, and fat levels from 2.3% to 2.93% [9,10]. These values highlight its potential as a functional ingredient for enhancing both macronutrient density and dietary fiber in cereal-based formulations. Globally, sorghum is the fifth most important cereal crop, cultivated extensively across Africa, Asia, and the Americas due to its drought tolerance and nutritional properties [11]. It is also gluten-free, making it suitable for individuals with celiac disease or gluten sensitivity. In Algeria, sorghum is traditionally cultivated in semi-arid inland regions and contributes to local food security, biodiversity preservation, and sustainable agriculture. Similarly, cork oak is a keystone species in Mediterranean ecosystems. Algeria hosts one of the largest cork oak forest covers in North Africa, where acorn harvesting is a culturally rooted and underutilized practice with substantial nutritional and functional food potential [12]. The reformulation of traditional pasta using locally available, non-conventional ingredients such as sorghum and cork oak flour responds to both nutritional and cultural drivers. Sorghum flour was chosen for its high content of dietary fiber and essential minerals (e.g., iron and magnesium), and cork oak acorn flour was chosen due to the presence of unsaturated fats and polyphenols.

The present study aims to develop a traditional Algerian pasta, *Trida*, enriched with sorghum flour (locally known as “draa” or “bechna”) (1%) and cork oak flour (locally called “ballout”) (4%). These substitution levels were selected based on internal pre-formulation tests (unpublished data; see Section 2.4) and are consistent with traditional uses reported in previous studies on fermented ingredients in couscous. In our previous study [12], we reported that traditional formulations typically incorporated fermented sorghum and acorns at levels ranging from 0.8% to 8%, with higher inclusion rates negatively affecting texture, color, or acceptability. To the best of our knowledge, this is the first study to evaluate the use of sorghum and cork oak flours in the reformulation of *Trida*, a traditional Algerian laminated pasta. While previous research has investigated the use of functional flours in conventional pasta types, the application of cork oak acorn flour in this context is virtually unexplored. By incorporating these local ingredients, this work seeks to diversify and enhance traditional pasta products, assess the feasibility of producing pasta with alternative flours, and evaluate the resulting products in terms of cooking behavior, texture, microstructure, and antioxidant potential compared to semolina-based controls. This work bridges the gap between traditional food heritage and modern nutritional demands by valorizing local biodiversity within a functional food framework. Moreover, these formulations yield a substantial reduction in digestible carbohydrates alongside a marked increase in dietary fiber, making them promising candidates for developing pasta products with improved glycemic responses. The dual focus on lowering glycemic impact and increasing fiber content responds to current consumer health trends and nutritional guidelines.

## 2. Materials and Methods

### 2.1. Reagents and Solvents

All solvents and reagents used were of analytical or HPLC grade. Methanol, ethanol, acetone, and acetonitrile were purchased from Sigma-Aldrich (St. Louis, Missouri, USA) and used for extraction procedures and chromatographic analyses. Formic acid (≥98%) and acetic acid were used to acidify mobile phases. DPPH (2,2-diphenyl-1-picrylhydrazyl), Folin–Ciocalteu reagent, sodium carbonate (Na_2_CO_3_), aluminum chloride (AlCl_3_), sodium nitrite (NaNO_2_), and sodium hydroxide (NaOH) were obtained from J.T. Baker (Phillipsburg, NJ, USA) and employed in antioxidant assays. Gallic acid and catechin (Sigma-Aldrich, St. Louis, MO, USA) were used as standards for total phenolic and flavonoid content determinations, respectively. For thin-layer chromatography, silica gel 60 F254 plates (Merck, Darmstadt, Germany) and 0.1% methanolic DPPH solution were used. Ultrapure water was obtained from a Milli-Q system (Millipore, Bedford, MA, USA) for all experimental procedures.

### 2.2. Raw Materials

Four commercial samples of durum wheat semolina (*Triticum durum*) were selected from the local Algerian market based on a market survey evaluating their suitability for pasta-making. Each was packaged in 25 kg bags and classified as “superior quality” (SSSE), typically employed in pasta production. The samples, labeled S1 (Spac), S2 (Kenza), S3 (Mexicali), and S4 (Bousbaa), were composed of 100% durum wheat with no added improvers or additives. S2 (Kenza) semolina emerged as the most commonly preferred by local producers for traditional pasta preparation due to its balanced technological characteristics. All samples were stored in sealed packaging under dry, ambient conditions until use. The particle size distribution of all semolina was determined by mechanical sieving [13], using a stack of sieves with mesh sizes of 500 µm, 315 µm, 180 µm, and 125 µm. For each sample, 100 g was sieved, and the retained fractions were recorded to characterize their granulation profiles. Semolina samples S1, S2, and S3 exhibited similar granulation, with most particles retained between 180 and 315 µm (S1–S3), whereas S4 exhibited a finer distribution, with increased particles <180 µm.

For enrichment, two whole flours were used: sorghum flour (SF) and cork oak fruit flour (CF). Both were purchased from local artisanal producers in the Jijel region (northeastern Algeria). These flours underwent traditional spontaneous fermentation at the producer level before commercialization. Fermentation is widely used in the region to reduce antinutritional factors such as tannins and phytic acid, which are naturally abundant in both sorghum and cork oak. The producers reported having performed fermentation using a traditional spontaneous process under ambient temperature. Sorghum was completely immersed in water for 6 to 8 months under aerobic or anaerobic conditions, depending on the substrate, as reported previously [11]. For cork oak acorns, the fermentation process typically lasts between 7 and 8 months, as reported by 100% of surveyed local producers [12]. The process improves not only the nutritional quality by enhancing mineral bioavailability but also the sensory properties by reducing bitterness. Particle size distribution of sorghum and cork oak flours was determined in our laboratory as for semolinas. Sorghum flour showed a broader particle size range predominantly between 315 and 500 µm, while cork oak flour was the coarsest, with a substantial portion retained above 500 µm.

The salt employed was extra-fine iodized table salt, intended for culinary use, and produced by the Algerian National Salt Company (E.N.A.Sel, Constantine, Algeria). Tap water, sourced from the municipal supply of Jijel, was used for dough hydration. According to local utility reports, this water is classified as moderately hard (~20–25 °F, French degrees) and features a neutral to slightly alkaline pH (~7.2–7.8), compliant with national potable water standards.

### 2.3. Chemical Composition of Raw Materials

The chemical composition of the semolina and the enrichment flours (sorghum and cork oak) was determined in triplicate using standardized laboratory methods to assess their suitability for pasta production.

Moisture and ash contents were analyzed according to AACC methods [14,15]. Total protein content was determined by the Kjeldahl method (ISO 20483:2006) [16]. Crude fat was analyzed following the Soxhlet extraction method (AOAC 920.39) [17], while total dietary fiber was measured using the enzymatic–gravimetric procedure (AOAC 985.29) [18]. Digestible carbohydrates were calculated by difference, and energy values were estimated using Atwater factors (4 kcal/g for proteins and carbohydrates; 9 kcal/g for lipids).

### 2.4. Pasta Preparation

Trida pasta samples were prepared using four different durum wheat semolinas (T1–T4), salt, and tap water. Kenza semolina (T2) was used as the control for enrichment trials. Two additives were employed for enrichment: 1% sorghum flour (SF) was added to T2 to obtain the enriched TSF pasta, while 4% cork oak flour (CF) was added to T2 to obtain the enriched TCF pasta. Specifically, enriched flour mixtures were produced by mixing 10 g of SF or 40 g of CF with 990 g or 960 g of semolina T2, respectively, to prepare 1 kg batches, as reported in Table 1. Salt (1%, corresponding to 10 g per 1 kg of flour mixture) was included in all formulations, and water addition was adjusted to 450 mL for TSF and 600 mL for TCF, compared to 400 mL for the control samples (T1–T4). The substitution levels were selected based on a series of internal pre-formulation tests, which evaluated pasta texture, integrity, and sensory acceptability at increasing concentrations (1–10%) of both SF and CF. Levels above 4% CF led to excessive darkening and grittiness, while levels above 1% SF negatively affected dough cohesiveness and cooking loss. Therefore, 1% and 4% were identified as the maximum inclusion levels that allowed technological feasibility and consumer acceptability and were selected for further characterization.

The dough was prepared by manually mixing all ingredients until complete semolina hydration (~5 min), followed by a 10 min resting period. Mechanical kneading was then performed using a Kenwood Chef XL stand mixer equipped with a dough hook for 10 min (8 min at low speed and 2 min at medium speed) to ensure proper development of the dough structure. The amount of added water varied across formulations according to the specific hydration needs of the flour blends: 400 mL for the control, 450 mL for TSF, and 600 mL for TCF.

After kneading, the dough was covered with a damp cloth and rested for 15 min at room temperature. It was then processed using the manual pasta roller Marcato Atlas 150 (Imperia, Sant’Ambrogio di Torino, Italy) and gradually reduced to a final sheet thickness of approximately 1.5 mm, which was verified with a digital caliper. The dough sheets were lightly dusted with corn flour to prevent sticking and manually cut into uniform square shapes (~1.5 × 1.5 cm) using a standardized metal grid cutter to ensure consistent dimensions. The pasta pieces were arranged in a single layer on stainless steel trays and dried under passive air circulation at room temperature (22 ± 2 °C) for up to 72 h. Drying was conducted in a clean, food-grade facility under hygienic conditions. The low water activity achieved during drying further prevented microbial proliferation, in accordance with HACCP protocols. Drying was conducted during late spring (May–June), under natural relative humidity conditions (60–65%) without active humidity control. The drying process was considered complete when a stable weight was recorded across two consecutive measurements taken 24 h apart. Dried samples were then stored in airtight containers at room temperature until further analysis.

### 2.5. Pasta Characterization

#### 2.5.1. Measurement of the Length, Width, and Thickness of Pasta

Pasta pieces were cut using a standardized mold to ensure uniform shape. Geometrical dimensions (length, width, and thickness) of the pasta samples were measured using a digital caliper, following standard metrological practices; length was taken as the longest axis [19]. For each pasta sample, twenty pieces were measured (n = 20).

#### 2.5.2. Determination of Water Absorption Index and Water Solubility Index

The water absorption index (WAI) and water solubility index (WSI) were measured in triplicate following the procedure described by Wójtowicz and Mościcki [20]. Dried uncooked pasta samples were ground using a laboratory-scale blade mill (IKA A11 basic, IKA-Werke GmbH & Co. KG, Staufen im Breisgau, Germany) to obtain a homogeneous powder. The resulting powder was then sieved through a 500 µm mesh to standardize the particle size before analytical procedures. In test tubes, 7 mL of distilled water was added to 0.7 g of ground dry pasta (a particle size less than 300 µm). After standing for 5 min, the sample was mixed for 10 min and then centrifuged at room temperature at 21,000× *g* for 10 min in a Digicen 21 laboratory centrifuge (Labsystem, Kraków, Poland). The supernatant was collected and dried in an oven at 110 °C to constant weight. The water absorption index and water solubility index were calculated using the following equations:

WAI = (weight of sediment)/(weight of dry solids)(1)

WSI = (weight of dissolved solids in supernatant × 100)/(weight of dry solids)(2)

### 2.6. Pasta Cooking Quality

Cooking behavior is determined through three main parameters: optimum cooking time (OCT), water absorption capacity (WAC), and cooking loss (CL). These indicators reflect the technological behavior of pasta during cooking and are commonly used to evaluate product performance and consumer acceptability. All analytical determinations were performed in triplicate.

#### 2.6.1. Optimum Cooking Time

The optimum cooking time (OCT) was determined according to the AACC International Approved Method 66-50 [21], with modifications from Chillo et al. [22]. A 10 g sample of flat pasta cut into small square strands (approximately 12–14 mm in length and 14 mm in width) was immersed in 300 mL of boiling distilled water. At regular 30 s intervals, a strand was removed and compressed between two Plexiglas plates to detect the presence of a white core, which indicates ungelatinized starch. The optimum cooking time (OCT) was recorded when the white core completely disappeared, indicating full gelatinization of the starch.

#### 2.6.2. Water Absorption Capacity

Water absorption capacity (WAC) was determined according to the AACC International Method [21]. A 10 g sample of pasta was cooked in 300 mL of boiling distilled water for the optimal cooking time, then rinsed with 100 mL of cold water (20 °C), and drained for 3 min using a strainer. The weight of the cooked pasta was recorded, and water absorption was calculated using the following equation:

WAC (%) = (weight of cooked pasta−weight of dry pasta/weight of dry pasta) × 100(3)

#### 2.6.3. Cooking Loss

Cooking loss (CL) was determined following the same method [21] using the procedure adapted from Wójtowicz and Mościcki [20]. Cooking loss, expressed as grams of dry matter lost per 100 g of dry pasta, was calculated as follows:

CL (%) = (weight of residue/weight of dry pasta) × 100(4)

### 2.7. Texture Profile Analysis

Texture measurements of cooked pasta for OCT and drained pasta (50 g) were performed using the Zwick-Roell BDO-FB0.5 TH instrument (Zwick GmbH & Co., Ulm, Germany), following the AACC International Approved Method 66-50.01 (Pasta and Noodle Cooking Quality—Firmness) [21]. A double-compression test was carried out using an Ottawa Texture Measuring System (OTMS) cell at a crosshead speed of 100 mm/min, applying 50% compression of the cooked pasta layer with a 10 s interval between cycles. The testXpert^®^ 13.3 software automatically calculated key textural parameters, including hardness, cohesiveness, gumminess, springiness, chewiness, and adhesiveness.

### 2.8. Color Profile Evaluation

Before color analysis, pasta samples were cooked in boiling water (100 °C) using a pasta-to-water ratio of 1:10 (*w*/*v*), following the optimum cooking time (OCT) previously determined for each formulation. After cooking, samples were rapidly cooled under running tap water, gently blotted dry with absorbent paper, and immediately analyzed. Absolute color measurements of cooked pasta samples were performed using Lovibond CAM-System 500 (The Tintometer Ltd., Amesbury, UK). The colorimetric parameters L* (lightness, where 0 = black and 100 = white), a* (red–green axis, with positive values indicating redness and negative values indicating greenness), and b* (yellow–blue axis, with positive values indicating yellowness and negative values indicating blueness) were quantified for each sample using the 2° standard observer position [23]. The instrument was calibrated prior to each analysis with certified white and black standard tiles to ensure measurement accuracy. The measurements were performed ten times for each pasta sample. To quantify the overall color difference between samples, the ΔE* was calculated according to the formula applied by Romankiewicz et al. [24], as follows:

(5) $$∆E=\sqrt{\;{\left(L_{sample}^{*}\;\;-\;\;L_{contol}^{*}\;\right)}^{2}+{\left(a_{sample}^{*}\;-\;a_{contol}^{*}\;\right)}^{2}+{\left(b_{sample}^{*}\;-\;b_{contol}^{*}\;\right)}^{2}}$$

### 2.9. Functional Components and Antioxidant Activity Analyses of Raw Materials and Pasta Products

The analyses were conducted on all flours (the four semolinas, sorghum flour, and cork oak flour) and on dried, uncooked pasta (T2, TSF, and TCF). All analyses were performed on uncooked pasta samples (T2, TSF, and TCF) to isolate the contribution of the added flours prior to cooking-induced degradation or leaching of bioactive compounds.

#### 2.9.1. Accelerated Solvent Extraction (ASE)

Extraction was performed on four semolinas (S1–S4), sorghum flour (SF), cork oak flour (CF), T2, and enriched pasta samples (TSF and TCF), all in a dried, uncooked form. Samples were ground using a laboratory mill (EGK, Rommelsbacher ElektroHausgeraete GmbH, Dinkelsbühl, Germany) with a 0.5 mm ring sieve to achieve homogeneous particle size. For each sample, 1.0 g of powder was weighed on an analytical balance and transferred to a 10 mL stainless steel extraction cell. Accelerated solvent extraction (ASE) was carried out using ethyl acetate, dichloromethane, methanol, 1% tartaric acid in methanol, and cyclohexane as solvents, under the following conditions: 80 °C, 111.5 bar, 10 min per cycle, with three successive cycles per solvent [25]. After evaporation to dryness, each extract was combined with methanol in a volumetric flask to reach 10 mL. All extractions were performed in triplicate.

#### 2.9.2. Qualitative Analysis of Selected Polyphenolic Compounds by High-Performance Liquid Chromatography Coupled to a Mass Spectrometer (HPLC/ESI-TOF-MS)

Extracts of sorghum (SF) and cork oak (CF), obtained as described in Section 2.9.1, were analyzed by high-performance liquid chromatography coupled with a mass spectrometer (HPLC/ESI-TOF-MS), using an Agilent G3250AA LC/MSD TOF system (Agilent Technologies, Santa Clara, CA, USA) equipped with an HP 1200 chromatograph, LC/MSD 6210 TOF mass spectrometer, autosampler, pump, degasser, and nitrogen generator. Separation was achieved on a Zorbax STABLE BOND RP-18 column (250 × 21 mm, 5 µm) at 25 °C using a gradient elution of solvent A (1% acetonitrile in water, 0.1% formic acid, 10 mM ammonium formate, pH 3.5) and solvent B (95% acetonitrile with the same additives). The gradient increased from 1% to 60% B over 45 min, reached 90% in the next minute, and was held for 4 min, with a flow rate of 0.2 mL/min and an injection volume of 10 µL. Analyses were performed in a negative ion mode at fragmentor voltages of 140, 200, and 250 V. Compounds were identified by high-resolution ESI-TOF-MS spectral analysis [26]. All analyses were performed in triplicate on independently prepared extracts.

#### 2.9.3. Determination of the Total Content of Polyphenolic Compounds

The total polyphenol content (TPC) in extracts from four semolinas (S1–S4), T2, two enrichment flours (SF and CF), and enriched pasta samples (TSF and TCF) was determined spectrophotometrically using the Folin–Ciocalteu (F–C) method [27]. A Perkin-Elmer A 15 UV/Vis spectrophotometer (Waltham, MA, USA) was used. A gallic acid calibration curve (0–1.0 mg/mL) was prepared by reacting known concentrations with the F–C reagent and sodium bicarbonate, followed by incubation at 40 °C for 50 min. Absorbance was recorded at 765 nm, and distilled water was used as a reference. TPC in the extracts was calculated as mg gallic acid equivalents (GAE)/mL. Blank samples (methanol instead of extract) were included for correction. Each determination was carried out in triplicate using independently prepared extracts.

#### 2.9.4. Determination of Total Flavonoid Content

Total flavonoid content (TFC) in extracts of semolinas (S1–S4), enrichment flours (SF and CF), T2, and enriched pasta samples (TSF and TCF) was determined by a colorimetric method using aluminum chloride [28]. A quercetin calibration curve (0–3.2 mg/mL) was prepared in methanol. Each sample (0.5 mL extract) was mixed with 5% NaNO_2_, 10% AlCl_3_, and 1 M NaOH and then diluted to 10 mL with distilled water. After 10 min, absorbance was measured at 510 nm using a UV-Vis spectrophotometer, with distilled water as the blank. Flavonoid concentration was expressed as mg quercetin equivalents (QE)/mL. Blank samples (methanol and water instead of extract and AlCl_3_) were prepared for baseline correction. All analyses were performed in triplicate on independently prepared extracts.

#### 2.9.5. Spectrophotometric Analysis of the Obtained Extracts to Determine Their Antioxidant Properties

The antioxidant activity of the extracts from semolinas (S1–S4), enrichment flours (SF and CF), T2, and enriched pasta samples (TSF and TCF) was determined using a spectrophotometric DPPH radical-scavenging assay [27]. A DPPH methanolic solution (0.001 mM) was prepared by dissolving 0.0039 g of DPPH in 100 mL of methanol and refrigerating overnight. Measurements were performed using a UV-Vis spectrophotometer at 517 nm. For each test, 2.5 mL of the DPPH solution was mixed with 0.5 mL of the extract, and absorbance changes were recorded at regular intervals until stabilization. A control sample (DPPH + methanol) and a blank (methanol only) were included. All determinations were carried out in triplicate on independently prepared extracts.

#### 2.9.6. TLC-DPPH Test of the Obtained Extracts

The antioxidant activity of the extracts was further assessed using a thin-layer chromatography (TLC)-based DPPH assay [29]. Silica gel plates (10 × 10 cm) served as the stationary phase, while the mobile phase consisted of ethyl acetate, toluene, and formic acid (10:10:0.5 *v*/*v*/*v*). Extracts (7 µL) were applied with an automated TLC applicator (Desaga AS-30) and developed unidirectionally. After drying, plates were sprayed with 0.1% methanolic DPPH solution and scanned after 0, 10, and 30 min. Images were processed using Sorbfil TLC Videodensitometr v 2.3 software (Sorbpolymer Ltd., Krasnodar, Russia), and antioxidant potential was expressed relative to semolina S2 extract activity (reference = 1). Analyses were conducted in triplicate.

### 2.10. Microstructure Observations

Samples of dry pasta and cooked products after freeze-drying were used to observe the microstructure. To prevent structural damage and preserve the morphology of the cooked samples, they were rapidly frozen by immersion in liquid nitrogen prior to freeze-drying. Pasta samples were mounted on aluminum specimen stubs using a double-sided adhesive silver tape to expose the surface and cross-section. Samples were sprayed with gold using the Emitech K550X Sputter Coater (Emitech, Essex, UK). The microstructure of pasta was observed with various magnifications of ×200 and ×600 with the scanning electron microscope Vega Tescan LMU (Tescan, Brno, Czech Republic) at an accelerating voltage of 20 keV [20].

### 2.11. Statistical Analysis

All experimental data are presented as mean ± standard deviation (SD) based on multiple measurements. Statistical analysis was performed using SPSS software (version 17.0, SPSS Inc., Chicago, IL, USA). A one-way analysis of variance (ANOVA) followed by Tukey’s HSD post hoc test was used to assess significant differences between means at *p* < 0.05. Different superscript letters in tables indicate statistically significant differences among samples.

## 3. Results and Discussion

### 3.1. Results of Chemical Composition of Raw Materials

The chemical composition analysis results of the raw materials in samples S1 (semolina Spac), S2 (semolina Kenza), S3 (semolina Mexicali), S4 (semolina Bousbaa), SF, and CF are tabulated in Table 2. The presented results revealed significant nutritional variability that aligns with their botanical origins, as well as with established literature values. The semolina samples (S1–S4), derived from durum wheat, exhibited higher protein content consistent with semolina values (~12–13% protein), supporting their suitability for products requiring strong dough structure, such as pasta.

In line with Gopalan et al. [30], semolina exhibited higher protein content compared to SF and CF, reflecting their distinct botanical origins. CF showed notably elevated fat content (8.69%) and energy value, likely due to its rich lipid composition (Table 2), while SF was characterized by the highest ash and fiber content (28.27%), confirming sorghum’s well-documented mineral richness and dietary fiber levels [9,10]. Moisture content was relatively consistent among semolina samples but lower in SF and CF, favoring an extended shelf life [31]. Carbohydrates were abundant in semolina (66.67–68.51%) but lower in SF and CF (48.49% and 45.41%), corresponding to their higher fiber and fat content [32,33]. These results align with the compositional profiles of whole-grain and fiber-enriched flours and are consistent with values reported for semolina and wheat flour products [34].

Importantly, the observed variations in fiber and lipid content support the use of each flour in health-oriented formulations. Such compositional traits are especially relevant for designing products targeting metabolic health and gut microbiota modulation [35]. Although CF exhibited a caloric content comparable to semolina, differences in starch digestibility may affect its actual energy availability. SF had a slightly lower caloric value, but its overall impact on the enriched pasta’s energy density remains limited due to its low inclusion level (1%). These considerations suggest that the tested additives may contribute to the development of functional foods with modulated caloric density, potentially supporting weight management strategies [36].

### 3.2. Pasta Characterization Results

#### 3.2.1. Geometric Dimensions of Pasta Samples

The results corresponding to the geometric dimensions of developed *Trida* pasta samples, including length, width, and thickness (in mm), are presented in Table 3.

Table 3 shows that TCF (pasta enriched with cork oak flour) exhibited the greatest average length, longer than control samples T1–T3, but statistically comparable to T4 and TSF. In terms of width, TCF was again wider than all semolina-based samples, while T2 and T3 had the narrowest widths. For thickness, T1 was the thickest, followed by TCF and T2, while TSF had the lowest thickness.

The dimensional analysis of pasta samples (Table 3) shows minimal variation across formulations, and an ANOVA confirmed that these differences were not statistically significant (*p* > 0.05). These measurements are therefore presented primarily for descriptive purposes, as they are unlikely to have influenced cooking behavior or texture. The slight variations observed in mean length, width, or thickness are within normal processing variability. Since T2 shares the same semolina base (S2) as the composite formulations TSF and TCF, it remains the most appropriate reference for other technological comparisons in this study.

#### 3.2.2. Water Absorption Index (WAI) and Water Solubility Index (WSI) Results of Pasta

The moisture content of *Trida* pasta presented in Table 4, prepared from six different semolina and flour types, shows no significant differences (*p* > 0.05), ranging from 10.89% (T1) to 11.47% (T2), with all samples falling within a narrow interval. These values are well within the international standards for dried pasta, which typically recommend a maximum moisture content of 12.5% to ensure product stability and safety [37]. The slight variations observed between samples may be attributed to differences in raw material composition or minor fluctuations in processing and drying conditions [38]. The consistently low moisture levels across all pasta samples indicate effective drying and suggest that each formulation is suitable for extended shelf life and resistant to microbial spoilage, as moisture content directly influences water activity, a critical factor for microbial growth [39]. Although WAI and WSI are more commonly applied to flours or extrudates, they were measured here on ground dried pasta to evaluate the intrinsic water interaction capacity of the pasta matrix, regardless of shape and structure. This approach was chosen to provide a comparative analysis of how enrichment with sorghum or cork oak flour alters hydrophilic behavior and solubility at the macromolecular level. While these indices do not directly correspond to cooking behavior, they contribute to understanding formulation effects on hydration potential.

Furthermore, WAI and WSI are important functional parameters reflecting the ability of starch and protein fractions in pasta to absorb and solubilize in water, respectively, which affect cooking quality and texture [40]. Although no statistical correlation analysis was performed in this study, the previous literature suggests that starch damage, protein content, and fiber levels may influence WAI and WSI values, and likely contribute to the trends observed [41], impacting water uptake during cooking and final product acceptability. The water absorption index (WAI) values for all samples (T1 to T4, TCF, and TSF) are very similar, ranging from 1.54 to 1.61%, indicating comparable water retention and hydration capacities across the samples. This suggests that the starch and protein matrix in the pasta samples has a similar capacity to absorb water, which is a key factor influencing cooking quality and texture [38]. The WSI instead showed greater variation: most samples have moderate WSI values between 5.81% and 7.33%, reflecting similar levels of soluble components such as degraded starch and soluble fibers [40]. The TSF sample exhibited a significantly higher and more variable WSI, suggesting greater release of soluble substances. This elevated solubility can be attributed to a higher content of damaged starch or soluble fiber fractions in the additive used, which may increase nutrient leaching and alter texture during cooking [41]. Higher WSI values are often correlated with softer texture and increased cooking loss, which could negatively impact sensory quality, but may also enhance the bioavailability of some nutrients [42]. Therefore, the distinct WSI profile of TSF implies potential differences in cooking behavior and functional properties compared to other samples, which should be considered when developing pasta products with specific texture or nutritional goals.

#### 3.2.3. Results of Pasta Color Profile

Brightness and yellowness are among the most important features of durum semolina pasta, valued by both consumers and manufacturers. Semolina pasta typically features a bright yellow color, which indicates high quality [43]. However, enrichment with alternative flours often alters the visual attributes. Table 5 shows the values of the cooked pasta colorimetric parameters.

Statistical analysis revealed significant differences among samples for all color parameters. Semolina-based pastas (T1 to T4) exhibited high lightness values, low redness, and moderate yellowness, which is consistent with the typical color profile of durum wheat pasta reported in previous studies [44,45,46]. Among these, T4 showed the highest lightness and yellowness, while T3 displayed lower b* values and L* values closer to the mean, which is related to a paler appearance. The cork oak flour-enriched pasta (TCF) had significantly lower lightness and higher redness, confirming its darker appearance (*p* < 0.05), likely due to the presence of phenolic compounds [12,47]. Interestingly, its b* values (yellowness) remained statistically similar to those of semolina pasta samples, although this may reflect overlapping chromatic contributions from brown pigments rather than a true preservation of carotenoids. The sorghum flour-enriched pasta (TSF) showed significantly lower b* values than all semolina pastas (*p* < 0.05), consistent with the influence of sorghum’s pigmentation profile [48]. Total color difference (ΔE) was used to evaluate overall visual variation. While differences among semolina pasta samples were statistically insignificant, both TSF and TCF had ΔE values of 3.70 and 14.20, respectively, confirming that enrichment resulted in visually noticeable changes compared to the reference (T1). Values above 3.50 indicate visual differences as reported by Romankiewicz et al. [24]. These results confirm that flour composition significantly influences pasta color, an essential quality parameter for consumer acceptability. Although semolina provides the classic bright yellow color, enrichment with cork oak or sorghum flours yields novel color characteristics and may appeal to consumers seeking functional or whole-grain products despite darker tones [43,49,50].

### 3.3. Cooking Behavior of Pasta

The cooking behavior of Trida pasta samples (Table 6) revealed significant variations in optimum cooking time (OCT), water absorption capacity (WAC), and cooking loss (CL), reflecting differences in formulation and microstructure.

The enriched pastas TSF (sorghum flour) and TCF (cork oak flour) showed the most distinct behavior compared to the control pasta (T2).

TCF exhibited the longest cooking time (2.30 min), followed by TSF (2.15 min), compared to the shorter and consistent OCTs of T1–T3 (2.00 min) and the particularly short value of T4 (1.30 min). The extended cooking times of TSF and TCF can be attributed to their higher fiber content, which increases water retention and delays starch gelatinization, an effect previously reported in fiber-enriched or legume-based pasta formulations [51]. Regarding water absorption, T2 displayed high WAC (253.69%), but this was exceeded by T4 (299.82%), suggesting a highly porous matrix likely due to smaller particle size and reduced gluten strength or altered starch properties [52]. In contrast, TCF had the lowest WAC (214.33%), indicating a denser internal structure, while TSF (236.88%) was closer to T3 (233.04%) and below T2. These findings highlight how flour type and substitution influence water uptake capacity. Cooking loss (CL) values remained below the 8% threshold typical for acceptable pasta quality. TSF (7.70%) and TCF (7.58%) were comparable or slightly better than T2 (7.90%), suggesting good cooking stability despite flour enrichment. T4 had the highest CL (8.08%), while T3 and TCF had the lowest values, further supporting the idea of a more cohesive starch–protein network in these samples [53]. Although no SEM analysis was conducted during the cooking process, the functional effects observed support the hypothesis that fiber-rich flours can enhance structural integrity through tighter starch–protein matrices. Overall, the incorporation of sorghum and cork oak flours significantly modified cooking behavior compared to T2 and fit within the variability observed among commercial semolina pastas (T1, T3, and T4), confirming the technological feasibility of these functional ingredients.

### 3.4. Texture Profile of Cooked Pasta

The texture of cooked pasta is a crucial factor influencing consumer acceptance, as it directly affects the eating experience and overall product appeal [54]. Table 7 presents the texture profile of semolina pasta and developed enriched pasta samples, highlighting differences in parameters such as hardness, adhesiveness, cohesiveness, and chewiness that can impact sensory perception and preference. T4 pasta exhibited the lowest hardness, gumminess, and chewiness, probably due to the finer semolina granulation and softer structure after cooking. In particular, the semolina (S4) used in T4 showed a higher proportion of fine particles (<180 µm) compared to the other samples, which may have contributed to a less compact and cohesive pasta matrix, affecting its mechanical resistance after cooking.

**Figure 1:**
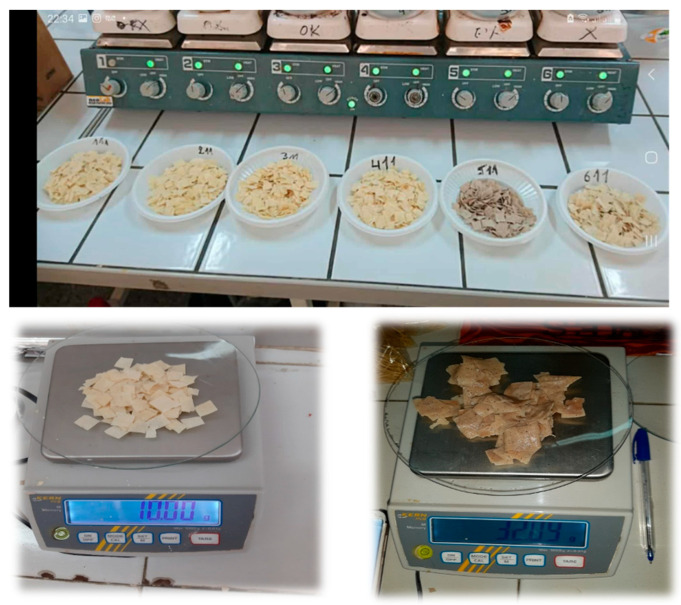
Visual comparison of dry pasta samples, T1, T2, T3, T4, TCF, and TSF (from left to right), alongside the TSF sample before and after cooking. This image illustrates both the baseline appearance of each formulation and the hydration-related transformation of TSF pasta after cooking.

Among the evaluated samples, TCF exhibited the highest values for hardness, gumminess, and chewiness, significantly exceeding all other formulations (*p* < 0.05), including the control T2 hardness. TSF, although softer than T2, retained comparable gumminess and chewiness values to T3 and showed improved texture over T4, which had the lowest structural resilience. Notably, springiness remained statistically similar across all samples, indicating that flour substitution did not impair the pasta’s elastic recovery. These results reinforce the role of ingredient composition and flour particle interactions in modulating post-cooking textural quality. The textural properties of the cooked enriched pasta samples demonstrate that incorporating cork oak flour (TCF) results in a pasta texture with significantly higher hardness, adhesiveness, cohesiveness, gumminess, springiness, and chewiness compared to traditional semolina pasta. In contrast, the sorghum flour-enriched pasta (TSF) showed moderate hardness and chewiness but lower adhesiveness and cohesiveness, likely due to its distinct protein and fiber profile that produces a softer, less elastic texture. All texture parameters of TSF are in the range or near those of the four semolina pastas, whereas TCF has constantly higher values. It seems harder, gummier, and chewier. These differences are the result of pasta composition: TCF has a higher substitution level than TSF, and it contains more fiber, more lipids, less protein, more TPC, and less TFC than TSF. According to Bolarinwa and Oyesiji [55], in their study about soy-fortified rice pasta, the increased gumminess observed in their pasta suggests that these samples are likely to have a sticky mouthfeel, which may be attributed to the high fiber content present in soy flour. To provide a visual comparison, Figure 1 presents the appearance of all dry pasta samples (T1–T4, TCF, and TSF) alongside the TSF sample before and after cooking, illustrating formulation-related differences and structural changes due to hydration.

### 3.5. Results of Functional Components and Antioxidant Activity of Raw Materials and Pasta Products

#### 3.5.1. Results of Qualitative Analysis of Selected Polyphenolic Compounds in Raw Materials by HPLC/ESI-TOF-MS

The HPLC/ESI-TOF-MS analysis of sorghum and cork oak flour extracts identified a diverse array of phenolic compounds, including flavonoids, phenolic acids, and condensed tannins. Notably, catechin, epicatechin, luteolin, and apigenin derivatives were detected in both flours, with a higher relative abundance in cork oak. The presence of gallic acid, caffeic acid, and ferulic acid derivatives highlights the antioxidant potential of the enrichment flours. These compounds are known for their ability to scavenge free radicals, corroborating the antioxidant results discussed in Section 3.5.3. These findings agree with previous studies emphasizing the complexity and richness of phenolic profiles in natural plant sources and their significance in functional food development [56].

**Figure 2:**
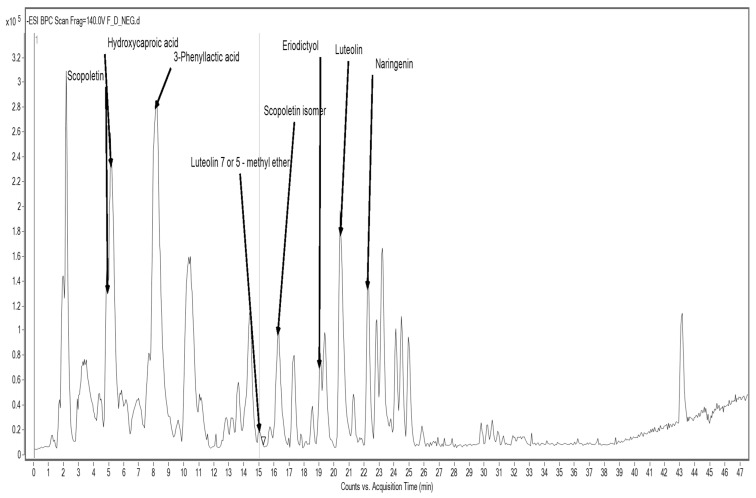
HPLC/MS chromatogram of ASE extract from sorghum flour SF.

The analysis of sorghum flour SF revealed a distinct phenolic fingerprint. Detected compounds included scopoletin and its isomer, hydroxycaproic acid, phenyl lactic acid, luteolin and its methyl ether, eriodictyol, and naringenin (Figure 2; Table 8).

Many of these compounds belong to the flavonoid subclass and exhibit a range of bioactivities. For instance, luteolin and naringenin are known for their anti-inflammatory, anti-cancer, and neuroprotective effects. Scopoletin, a coumarin derivative, has been reported to possess antioxidant and immunomodulatory activity, while eriodictyol has shown promise in mitigating oxidative damage and inflammation [57].

**Figure 3:**
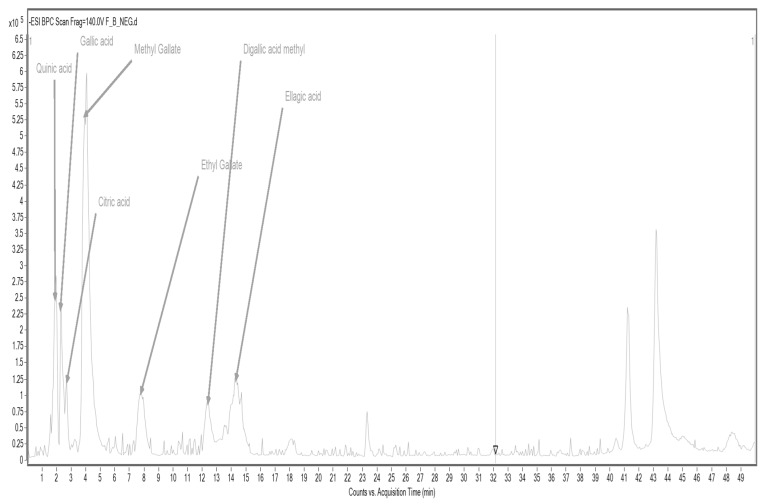
HPLC/MS chromatogram of ASE extract from cork oak flour CF.

In the case of cork oak flour CF, accelerated solvent extraction (ASE) allowed the identification of several key phenolic acids and their derivatives, including quinic acid, gallic acid, citric acid, ethyl gallate, methyl gallate, a methylated form of digallic acid, and ellagic acid (Figure 3; Table 9).

These compounds are known for their potent antioxidant, anti-inflammatory, and antimicrobial properties [58]. Gallic acid and ellagic acid are well-documented for their radical-scavenging abilities and protective roles against oxidative stress-related diseases, including cardiovascular and neurodegenerative disorders. Furthermore, the use of ASE proved effective in extracting a wide range of polar and semi-polar phenolic compounds, highlighting its suitability for profiling complex plant-based matrices. These detailed phenolic maps not only contribute to understanding the chemical diversity of functional food ingredients but also provide a scientific basis for future studies exploring their bioactivity and health-promoting effects.

#### 3.5.2. Results of Total Content of Polyphenolic Compounds (TPC)

Polyphenols are widely recognized for their health-promoting properties, particularly their ability to scavenge free radicals, modulate enzyme activity, and influence cell signaling pathways associated with inflammation [58,59]. The total polyphenol content expressed as gallic acid equivalents (GAE) varied significantly among the tested samples or raw materials, additives, and prepared pasta (Table 10).

The lowest TPC values were observed in flours, particularly in semolina, which is in line with previous findings that refined cereal products contain limited phenolic compounds due to the removal of the bran layer during milling [60]. Whole-grain flours tend to retain a broader spectrum of polyphenols; however, semolina, being highly refined, lacks much of this bioactive fraction. Pasta samples showed intermediate levels of TPC, likely reflecting the dilution of added ingredients [61]. The fortification of pasta with polyphenol-rich additives significantly enhanced its phenolic profile. Cork oak flour CF exhibited higher TPC than sorghum flour SF. This result agrees with the known phytochemical composition of cork oak flour, which is rich in hydrolysable tannins, phenolic acids, and flavonoids. The control pasta T2, made solely from Kenza semolina (S2), showed a TPC of 0.110 mg GAE/mL, confirming that enrichment with sorghum or cork oak significantly increases polyphenol levels compared to the unfortified pasta. Sorghum flour SF, while slightly lower in TPC than cork oak bark flour CF, still represents a valuable source of antioxidant polyphenols [62,63].

#### 3.5.3. Results of Total Flavonoid Content (TFC)

The analysis of total flavonoid content TFC, expressed as quercetin equivalents, revealed generally low levels in both semolina and pasta (Table 10). These findings are consistent with previous research indicating that traditional cereal-based products, particularly refined wheat flours such as semolina, are typically poor sources of flavonoids due to the removal of bran and germ during milling [60]. In contrast, the highest flavonoid concentrations were observed in the additives used—most notably in cork oak flour CF, followed by sorghum flour SF. CF flavonoid content is well documented in the literature; it is particularly rich in polyphenolic compounds such as flavonols, flavan-3-ols, and ellagitannins [7,8]. Sorghum SF also contributes significantly to the flavonoid profile, particularly through its high levels of 3-deoxyanthocyanidins, which are known for their antioxidant and anti-inflammatory properties [64]. Pasta T2 had a slightly lower total flavonoid content than pure semolina S2. Surprisingly, total flavonoid content in both enriched pasta types (TSF and TCF) was not higher, but it was slightly lower than in T2 pasta.

It seems that pasta production may have influenced this result. Flavonoids can bind to proteins or polysaccharides in the dough during kneading and forming. The main mechanisms of binding to proteins include the formation of hydrogen bonds between the hydroxyl groups of flavonoids and the carbonyl or amino groups of proteins (especially gluten) and hydrophobic interactions with the nonpolar regions of proteins. Polysaccharides present in sorghum, e.g., arabinoxylans, have the ability to form spatial networks in which flavonoids can be “trapped.” As a result, the following may occur: the formation of unstable complexes based on hydrogen bonds and van der Waals forces, the adsorption of flavonoids on the surface of fibers, and physical incorporation (mechanical closure in the gel structure). Flavonoids also have electron donor systems that easily bind metals (Fe^2+^, Mg^2+^, and Ca^2+^), forming metal–flavonoid complexes. The various types of complexes described above reduce the solubility of flavonoids, make them difficult to extract during analysis, and reduce their detectability in analysis (e.g., UV-Vis) [65]. Flavonoids are also susceptible to enzymatic and non-enzymatic oxidation, especially under conditions typical for manual pasta production (contact with oxygen and light). Enzymatic oxidation involves PPO (polyphenol oxidase), an enzyme naturally present in sorghum and other cereal plants, among others. It catalyzes the conversion of flavonoids to quinones, which are highly reactive and easily polymerize. Non-enzymatic oxidation occurs in the presence of oxygen from the air (auto-oxidative oxidation process), UV light (photo-oxidation), and transition metal ions [66].

#### 3.5.4. Results of Antioxidant Properties of Raw Materials and Pasta by Spectrophotometric Method Using DPPH

The results are presented as the degree of scavenging activity against DPPH, a stable free radical. (RSA %, Table 11).

The antioxidant capacity of the pasta samples reflects their total polyphenol content, as phenolic compounds are key contributors to radical-scavenging activity. The enriched formulations (TSF and TCF) showed a higher total polyphenol content compared to the control, which is consistent with the natural phenolic composition of sorghum and cork oak acorns. These compounds, including phenolic acids and tannins, are known for their strong antioxidant potential, which can enhance the nutraceutical value of pasta products. The increase in total polyphenol content likely explains the improved antioxidant activity observed in the enriched samples. This highlights the potential of incorporating plant-based ingredients as a strategy to develop functional pasta with added bioactive properties. Further studies could explore the stability of these phenolic compounds during processing and their bioaccessibility upon digestion [67].

#### 3.5.5. TLC-DPPH Results of the Extracts Obtained from Raw Materials and Pasta Products

**Figure 4:**
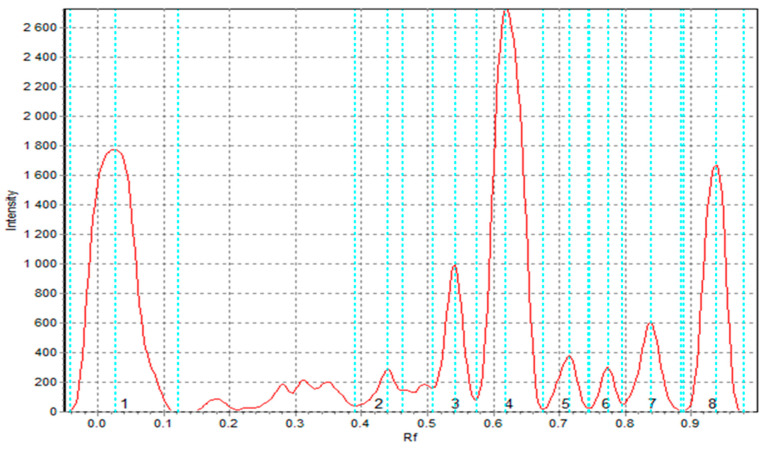
TLC chromatogram for sorghum flour extract (SF) processed with Sorbil TLC Videodensitometer v 2.3 software; scan after 30 min.

**Figure 5:**
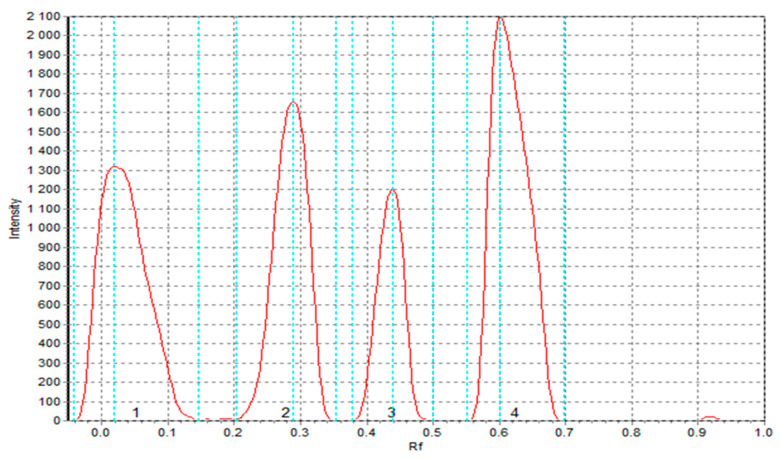
TLC chromatogram for cork oak flour extract (CF) processed with Sorbil TLC Videodensitometer software; scan after 30 min.

The results of TLC-DPPH radical scavenging in the tested extracts are presented in Table 12. Statistical analysis revealed significant differences (*p* < 0.05) in antioxidant activity among the samples at each time point. As shown in Table 12, CF extract exhibited the highest radical-scavenging activity at all time intervals, followed by SF, TCF, and TSF, with S2 consistently displaying the lowest activity. Eight peaks corresponding to antioxidant compounds were identified in the TLC chromatogram of SF extract (Figure 4), confirming its chemical richness. TSF extract displayed five peaks, indicating a partial loss of bioactive compounds during pasta processing. In contrast, the semolina extract (S2) showed only three low-intensity peaks, reflecting a limited antioxidant profile. CF and its pasta product (TCF) both exhibited four distinct peaks (Figure 5), confirming the contribution of cork oak phenolics to the antioxidant capacity of enriched pasta. These findings are consistent with spectrophotometric DPPH results, reinforcing the superior antioxidant potential of cork oak and sorghum flours. The control pasta T2 showed intermediate antioxidant activity (1.120 after 30 min), slightly higher than S2 semolina but significantly lower than TSF and TCF, confirming again the functional enhancement provided by enrichment.

The observed differences between raw flours and corresponding pasta products are likely because of oxygen and matrix interactions during formulation, which can influence the retention and accessibility of antioxidant compounds.

The results of the quantitative analysis of antioxidant compounds are consistent with the qualitative data. The highest values of area under the chromatographic peaks were obtained for CF flour, followed by TCF pasta, while the lowest values were observed in the S2 semolina sample. These findings highlight the effectiveness of CF substitution in enhancing the antioxidant profile of the pasta matrix. Despite the relatively low substitution level (4%), the TCF pasta exhibited a substantial increase in both total antioxidant capacity and polyphenol band diversity compared to the control (S2). Also, TSF has increased antioxidant activity, although a little less. This suggests that the enrichment effect was not merely additive or proportional to the concentration of the added ingredient but may also have been influenced by interactions within the pasta matrix or by increased extractability of polyphenols after processing.

These outcomes underscore the potential of CF, even at low inclusion levels, for the development of functional foods with enhanced health-promoting properties. To further support the phenolic composition analysis, TLC chromatograms of S2, TSF, and TCF were included as Supplementary Figures (Figures S1–S3), illustrating differences in band intensity and diversity across formulations. The observed differences in antioxidant profiles between whole-grain-derived samples and refined semolina are consistent with previous literature reports. As demonstrated by Durazzo et al. [60], refined wheat flours typically contain significantly reduced levels of phytochemicals, mainly due to the removal of seed coat layers during milling—layers that are primary sources of antioxidant compounds.

While this study focused on raw flours for antioxidant assessment, we acknowledge the importance of analyzing antioxidant activity in the final cooked product. Thermal processing may alter phenolic content through degradation or leaching, thereby influencing both nutritional and visual attributes. However, to isolate the specific contribution of each flour and minimize confounding factors such as cooking water absorption or processing-induced transformations, our present analysis was limited to uncooked matrices. Future work will investigate the stability and bioaccessibility of antioxidants in cooked pasta, with particular attention to retention during cooking and implications for consumer health.

### 3.6. Pasta Microstructure

**Figure 6:**
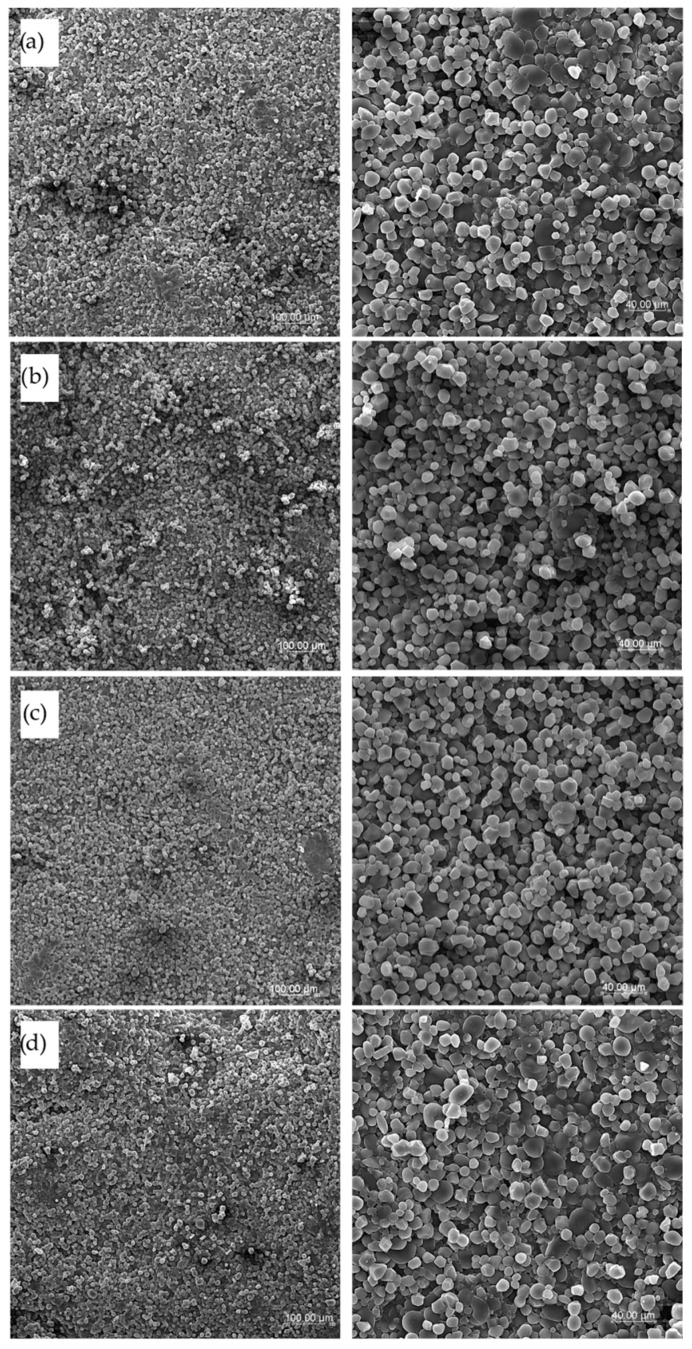
SEM pictures of dry pasta products’ surfaces with various magnifications (×200 left column and ×600 right column): (**a**) semolina T2 pasta, (**b**) semolina T4 pasta, (**c**) TSF pasta with sorghum flour addition, and (**d**) TCF pasta with cork oat flour addition.

**Figure 7:**
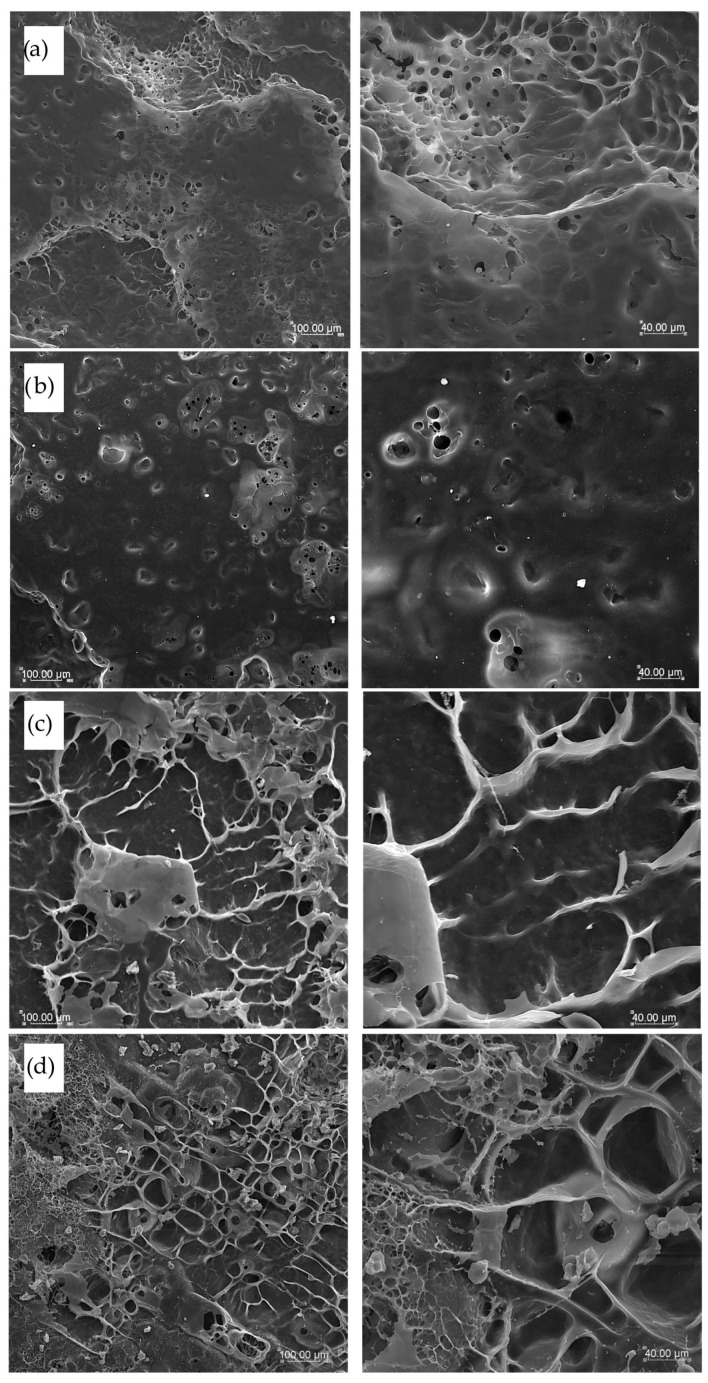
SEM pictures of cooked pasta products’ surfaces with various magnifications (×200 left column and ×600 right column): (**a**) semolina T2 pasta, (**b**) semolina T4 pasta, (**c**) TSF pasta with sorghum flour addition, and (**d**) TCF pasta with cork oat flour addition.

Structural pictures of the surface of the tested pasta were taken both for dry pasta as well as after cooking as freeze-dried samples at various magnifications (×200 and ×600). SEM surface pictures of dry products are presented in Figure 6, and the surface of cooked pasta products is shown in Figure 7. There are visible raw granules of semolina starch on the surface of dry pasta (Figure 6a,b) with a specific shape and size, with no significant differences between semolina pasta T2 and T4. Some places on the pasta surface are rough with a more uneven surface than observed for enriched pasta products, especially for T4 pasta. This may be an explanation for such high WAC results obtained for this pasta, where the cooking water had greater access to the pasta’s internal structures through the porous surface. If sorghum flour was applied in the recipe, the surface of *Trida* pasta was compact and smooth (Figure 6c), whereas pasta with cork oak flour addition showed a smooth surface with melted places where some bigger particles with different sizes and shapes were observed, indicating the presence of additives (Figure 6d), especially at high magnification (×600).

The surface of cooked and freeze-dried pasta products presented in Figure 7 showed a very compact and smooth surface in pasta T2 and T4 made with semolina only (Figure 7a and Figure 7b, respectively). This uniform and flat surface structure is characteristic of semolina pasta, whereas a flatter surface was noted for T4 pasta, where semolina with the finer particle size was used. Pasta TSF enriched with sorghum flour formed on the surface of some cooked agglomerates with visible longitudinal protein structures embedded in a molten starch matrix (Figure 7c) due to the high content of protein in sorghum flour added to pasta products. In this pasta, the most compact and firm structure was observed with the highest hardness, cohesiveness, and chewiness results. The most contrasting structure was the surface observed in TCF pasta (Figure 7d). Here, a lot of fibrous structures were found linked to each other with small empty spaces between, with low cohesiveness and springiness results of this pasta after cooking. In this pasta, minimal cooking time and WAC results were the lowest, making this pasta more dense and less elastic, probably due to the highest fiber content coming from cork oak flour addition.

**Figure 8:**
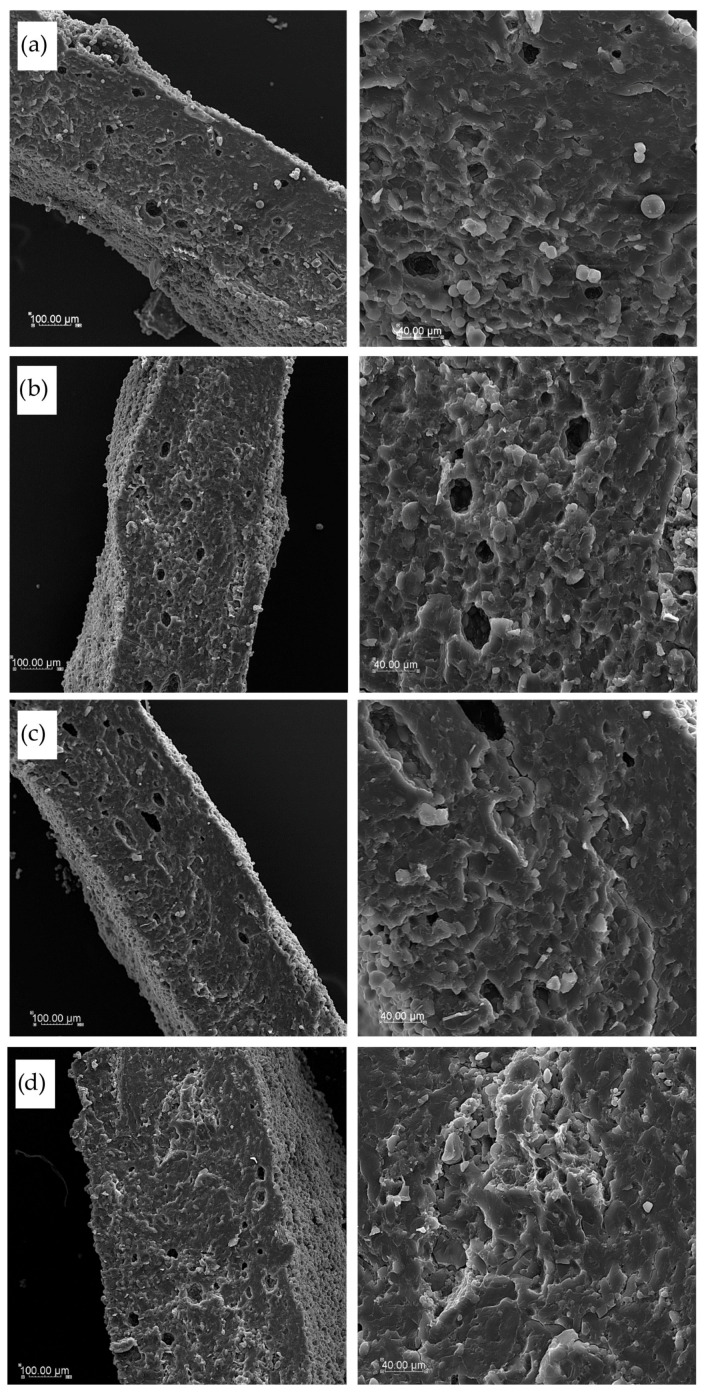
SEM pictures of dry pasta products’ cross-sections with various magnifications (×200 left column and ×600 right column): (**a**) semolina T2 pasta, (**b**) semolina T4 pasta, (**c**) TSF pasta with sorghum flour addition, and (**d**) TCF pasta with cork oat flour addition.

Figure 8 illustrates cross-section pictures of the tested dry pasta obtained with various magnifications by the SEM technique. The structure and thickness of T2 and T4 pasta were similar (Figure 8a and Figure 8b, respectively). A lot of singular starch granules were visible both on cross-section and surface, as well as a few empty spaces inside the dry pasta structures. A more dense and compact internal structure was found in the TSF cross-section with a lower thickness of pasta squares, confirming the results of geometric dimensions of dry pasta (Figure 8c). In this pasta, some starch granules were embedded into a uniform and smooth structure, which was more compact than in semolina pasta. Then, if a cork oak flour was used as an additive, the internal structure was compact but less uniform with visible agglomerates of raw materials banded with a fibrous matrix of protein and fiber from cork oak flour (Figure 8d).

**Figure 9:**
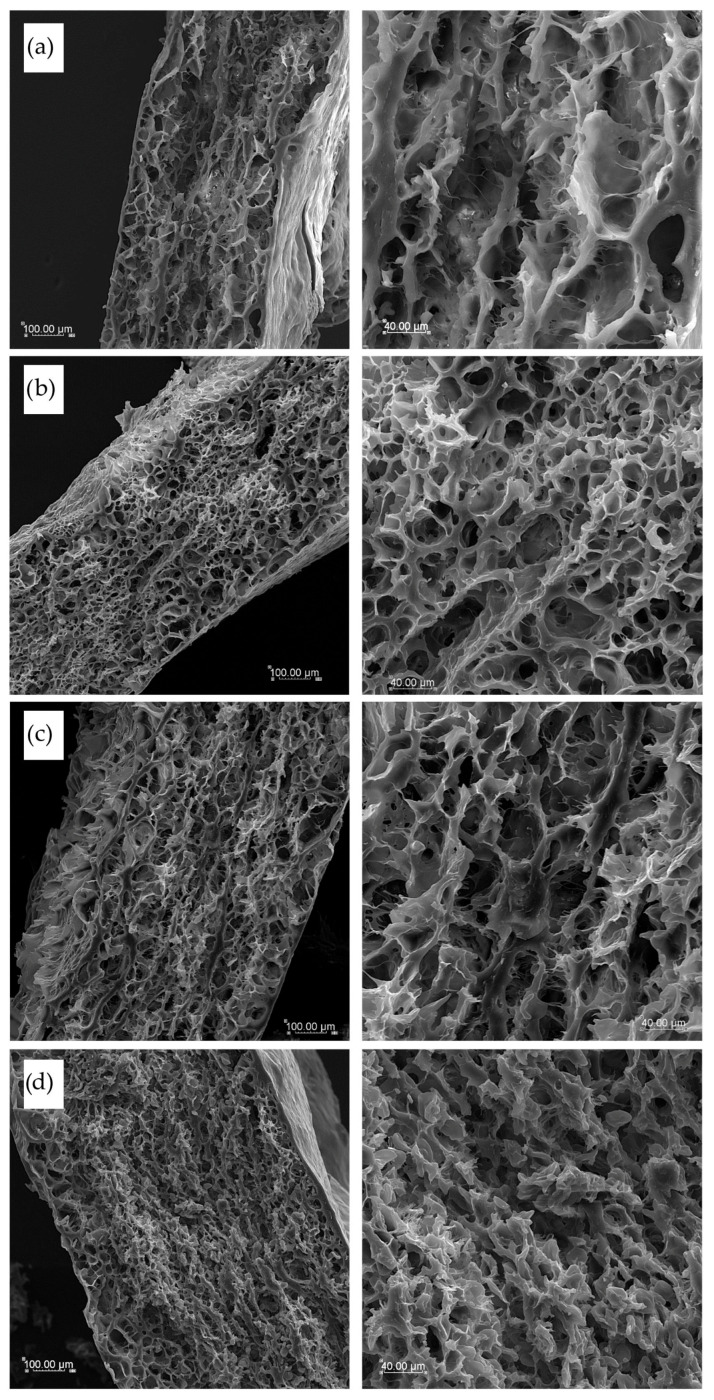
SEM pictures of cooked pasta products’ cross-sections with various magnifications (×200 left column and ×600 right column): (**a**) semolina T2 pasta, (**b**) semolina T4 pasta, (**c**) TSF pasta with sorghum flour addition, and (**d**) TCF pasta with cork oat flour addition.

Cooked pasta cross-section pictures, shown in Figure 9, differed depending on pasta components. T2 semolina pasta appeared to have a loose internal structure with big empty spaces after water penetration during cooking (Figure 9a). In T4 pasta, where the internal structure was more spongy-like, a clear protein network of gluten chains is visible, and small empty pores inside are more uniform in size (Figure 9b). Pasta enriched with sorghum flour, presented in Figure 9c, showed not only a gluten network but also some thicker and longer protein chains, as sorghum flour was rich in proteins other than gliadin and glutenin from durum semolina. These thick, dense, and long molten chains may be the reason for the highest hardness, cohesiveness, and chewiness results obtained for pasta texture in a double-compression test. This internal structure was leafy-like, and several layers were observed as formed during the hand rolling of the dough. TCF pasta products (Figure 9d) showed the smallest empty space inside, with very short internal connections between protein structures, probably due to the high fiber and fat content in cork oak flour. These could disturb the formation of a uniform long-chain structure after cooking, and a lot of small spaces with disrupted structure after leaching pasta components into cooking water were found in the inside structure of TCF pasta.

Romano et al. [68] investigated the microstructure of durum wheat semolina starch, identifying predominantly A-type granules with an average diameter of ~10 μm and smaller B-type granules averaging 2–3 μm. Lu et al. [69] confirmed the coexistence of A-type (diameter >9.9 μm) and B-type (<9.9 μm) granules in common wheat flour, reporting that A-type granules comprise approximately 70% of the starch volume but only 10% of the total granule count, while B-type granules account for 30% of the volume and 90% of the granules. Geera et al. [70] additionally described the presence of C-type granules (diameter <5 μm), usually classified within the B-type fraction, which contribute less than 3% of starch by weight in the wheat endosperm.

The comparative literature on sorghum flour (SF) reveals starch granules typically ranging from 1 to 10 μm, with polygonal to spherical morphology, embedded in a dense protein–starch matrix that limits granule isolation and swelling, as described by Haziman et al. [71]. In contrast, cork oak flour (CF), though less studied, has been reported to display irregular, amorphous starch-like structures, possibly resulting from its high content of polyphenols, fiber, and non-starch polysaccharides that interfere with typical granule morphology [72]. These differences in native starch structure among flours may explain the distinct hydration and gelatinization behavior observed in the enriched pasta samples and should be considered when formulating functional products with alternative plant-based ingredients.

In our developed dry pasta, mostly A-type granules are present, which are lenticular in shape, with a diameter of over 10 μm, but also, B-type granules are visible as small granules with a spherical shape. Martín-Esparza et al. [50] obtained a structure of uncooked pasta similar to ours, where the addition of high-fiber tiger nuts and xanthan produced a compact starch–protein matrix visible in cross-section, stabilizing the product and leaving only a few empty holes. In cooked pasta, the internal structure was similar to that reported by Gallo et al. [72] for spaghetti made from durum wheat semolina and whole-grain durum wheat semolina cooked spaghetti. They reported indistinguishable starch granules and protein matrices in the external part of pasta after cooking because of protein coagulation and starch gelatinization via the cooking process. The internal structure closer to the pasta core consisted of swollen starch granules embedded in a coagulated but dense protein network. This was only slightly observed in our pasta due to the shape and small thickness of pasta squares, which can be hydrated in a shorter time than spaghetti-shaped pasta, and does not contain a core part.

## 4. Conclusions

This study demonstrates the applicability of sorghum flours (*Sorghum bicolor*) and cork oak (*Quercus suber*) as enriching traditional Algerian *Trida* pasta in nutritional and functional compounds. These ingredients are readily available in Algeria through traditional farming or foraging practices and support biodiversity valorization, circular food systems, and alignment with global trends in sustainable and functional food innovation. Chemical composition analysis confirmed that semolina-based pastas offer higher protein content, while both cork oak and sorghum flours contributed to increasing the total dietary fiber. All pasta samples maintained low moisture content, which is related to product stability and shelf life. An assessment of physiochemical properties showed comparable water absorption across samples, with increased water solubility observed in sorghum-enriched pasta. Colorimetric evaluation revealed that flour type strongly influences pasta appearance, with semolina producing the brightest, most yellow pasta, sorghum flour addition resulting in less yellow products, and cork oak flour addition yielding darker and more red pasta. Cooking behavior varied in the tested samples, especially in WBC and texture profile, indicating the important effect of both sorghum flour and cork oak flour on the cooking behavior and structure of developed TSF and TCF pasta. TSF has color, cooking behavior, and texture more similar to those of common pasta (represented by T1, T2, T3, and T4), whereas TCF seems to be more different. HPLC/ESI-TOF-MS profiling confirmed a rich diversity of polyphenolic compounds in the enriched pasta, both those with sorghum and cork oak flours. Importantly, antioxidant analyses, including total polyphenol content and DPPH radical-scavenging activity, demonstrated that cork oak flour imparted the highest antioxidant capacity, followed by sorghum flour and semolina. These findings must be interpreted considering both the antioxidant content of the raw flours and their respective inclusion levels (1% and 4%), which influenced the magnitude of enrichment. The structural differences observed by SEM between enriched and control pasta samples were consistent with the cooking behavior and textural observations, supporting the hypothesis that flour type alters internal matrix architecture. These findings highlighted the potential of using underutilized local ingredients to produce nutritionally valid and functionally diverse pasta products, which may contribute to improved dietary antioxidant intake and help prevent diseases associated with oxidative stress. Nevertheless, this study presents certain limitations that should be addressed in future work. While the present analysis focused on raw materials and uncooked pasta to better assess the intrinsic antioxidant potential and polyphenol profiles, cooking processes may significantly impact these compounds through degradation, leaching, or structural transformation. Therefore, the antioxidant activity should also be investigated in cooked pasta to evaluate its real nutritional contribution. Furthermore, although HPLC-MS analyses confirmed the presence of polyphenolic compounds from TSE (1%) and TCF (4%) in the enriched formulations, their quantitative contribution post-cooking remains uncertain due to the low inclusion levels and possible thermal losses. Future studies should explore the retention and bioaccessibility of these bioactives in cooked products to clarify their impact on health-promoting properties. Finally, future investigations will include sensory acceptability studies on cooked pasta, evaluation of dose–response effects of flour enrichment, and assessment of the bioaccessibility and bioavailability of antioxidant compounds through in vitro digestion models and simulated gastrointestinal conditions. This will allow a more comprehensive validation of the nutritional functionality of the developed formulations.

## Figures and Tables

**Table 1:** Pasta formulations.

Sample Type	Semolina (g)	Enrichment Flour (g)	Salt (g)	Water (mL)
Control T1–T4	1000 (S1–S4)	0	10	400
Sorghum-Enriched TSF	990 (S2 Kenza)	10 (1%)	10	450
Cork Oak-Enriched TCF	960 (S2 Kenza)	40 (4%)	10	600

**Table 2:** Chemical composition analysis of the raw materials.

Sample	Moisture (%)	Protein (%)	Fat (%)	Ash (%)	Fiber (%)	Digestible Carbohydrates (%)	Energy (kJ)	Energy (kcal)
S1	11.56 ^b^	12.02 ^d^	1.44 ^d^	0.77 ^d^	5.70 ^c^	68.51 ^a^	1468	346
S2	11.95 ^a^	13.07 ^a^	1.52 ^d^	0.82 ^c^	5.97 ^c^	66.67 ^b^	1460	345
S3	11.07 ^c^	12.83 ^b^	1.48 ^d^	0.74 ^e^	5.96 ^c^	67.92 ^a^	1475	348
S4	11.09 ^c^	12.25 ^c^	1.83 ^c^	0.83 ^c^	6.19 ^c^	67.81 ^a^	1478	349
SF	8.96 ^e^	8.42 ^e^	3.17 ^b^	2.69 ^a^	28.27 ^b^	48.49 ^c^	1311	313
CF	9.60 ^d^	4.33 ^f^	8.69 ^a^	1.90 ^b^	30.07 ^a^	45.41 ^d^	1408	337

S1–S4—semolina; SF—sorghum flour; CF—cork oak flour. Values are expressed as mean ± standard deviation (n = 3). All analyses were performed in triplicate. Standard deviations were below 3% and are therefore not reported. Different superscript letters within the same column indicate statistically significant differences (*p* < 0.05).

**Table 3:** Dimension characteristics of *Trida* pasta samples: mean lengths, widths, and thicknesses.

Sample	Length (mm)	Width (mm)	Thickness (mm)
T1	13.31 ± 1.60 ^a^	13.25 ± 1.75 ^a^	0.81 ± 0.14 ^a^
T2	13.50 ± 2.00 ^a^	12.81 ± 1.96 ^a^	0.75 ± 0.07 ^a^
T3	13.45 ± 1.45 ^a^	12.95 ± 1.95 ^a^	0.66 ± 0.14 ^a^
T4	14.00 ± 1.00 ^a^	13.25 ± 1.25 ^a^	0.67 ± 0.13 ^a^
TSF	13.90 ± 1.40 ^a^	13.36 ± 1.83 ^a^	0.63 ± 0.12 ^a^
TCF	14.35 ± 1.15 ^a^	13.85 ± 1.35 ^a^	0.75 ± 0.03 ^a^

T1–T4: semolina pasta formulations; TSF: sorghum flour pasta; TCF: cork oak flour pasta. Values are expressed as mean ± standard deviation (n = 20). Different superscript letters within the same column indicate statistically significant differences (*p* < 0.05).

**Table 4:** Physiochemical characteristics of the raw pasta made with various semolina types and enriched with cork oak and sorghum flours.

Sample	Moisture Content (%)	WAI (%)	WSI (%)
T1	10.887 ± 0.014 ^d^	1.60 ± 0.02 ^a^	6.23 ± 0.16 ^c^
T2	11.468 ± 0.004 ^a^	1.57 ± 0.02 ^ab^	6.38 ± 0.45 ^c^
T3	11.203 ± 0.015 ^b^	1.60 ± 0.01 ^a^	6.62 ± 0.38 ^c^
T4	11.239 ± 0.021 ^ab^	1.61 ± 0.03 ^a^	5.81 ± 0.33 ^c^
TSF	10.905 ± 0.039 ^d^	1.54 ± 0.01 ^b^	10.19 ± 1.13 ^a^
TCF	11.179 ± 0.002 ^c^	1.57 ± 0.01 ^ab^	7.33 ± 0.22 ^b^

WAI—water absorption index; WSI—water solubility index; T1—T4—semolina pasta; TSF—pasta with sorghum flour; TCF—pasta with cork oak flour. Values are expressed as mean ± standard deviation (n = 3). Different superscript letters within the same column indicate statistically significant differences (*p* < 0.05).

**Table 5:** Colorimetric parameters of the cooked pasta made with various semolina types and enriched with cork oak and sorghum flours.

Sample	L*	a*	b*	ΔE
T1	59.95 ± 0.8 ^bc^	1.17 ± 0.3 ^b^	10.45 ± 0.7 ^a^	Ref
T2	61.14 ± 0.7 ^ab^	1.02 ± 0.4 ^b^	9.65 ± 0.6 ^a^	1.44
T3	60.33 ± 0.6 ^abc^	0.82 ± 0.3 ^b^	9.20 ± 0.5 ^a^	1.35
T4	61.42 ± 0.7 ^a^	0.85 ± 0.4 ^b^	10.64 ± 0.6 ^a^	1.52
TSF	57.74 ± 0.9 ^c^	1.25 ± 0.5 ^b^	7.48 ± 0.7 ^b^	3.70
TCF	46.20 ± 1.0 ^d^	4.71 ± 0.6 ^a^	10.59 ± 0.8 ^a^	14.20

L* = lightness; a* = redness; b* = yellowness; ΔE = total color difference (vs. T1); T1–T4 = semolina pasta; TSF = pasta with sorghum flour; TCF = pasta with cork oak flour. Values are expressed as mean ± standard deviation (n = 10). Different superscript letters within the same column indicate statistically significant differences (*p* < 0.05).

**Table 6:** Cooking behavior of *Trida* pasta prepared with various semolina types and enriched with cork oak and sorghum flours.

Sample	OCT (min)	WAC (%)	CL (%)
T1	2.00 ± 0.01 ^b^	241.99 ± 0.86 ^c^	7.85 ± 0.03 ^b^
T2	2.00 ± 0.01 ^b^	253.69 ± 1.08 ^b^	7.90 ± 0.02 ^b^
T3	2.00 ± 0.02 ^b^	233.04 ± 0.57 ^d^	7.58 ± 0.03 ^c^
T4	1.30 ± 0.01 ^c^	299.82 ± 0.12 ^a^	8.08 ± 0.04 ^a^
TSF	2.15 ± 0.17 ^ab^	236.88 ± 1.05 ^a^	7.70 ± 0.02 ^bc^
TCF	2.30 ± 0.05 ^a^	214.33 ± 1.23 ^e^	7.58 ± 0.03 ^c^

OCT—optimum cooking time; WAC—water absorption capacity; CL—cooking loss; T1—T4—semolina pasta; TSF—pasta with sorghum flour; TCF—pasta with cork oak flour. Values are expressed as mean ± standard deviation (n = 3). Different superscript letters within the same column indicate statistically significant differences (*p* < 0.05).

**Table 7:** Texture parameters of cooked pasta samples.

Sample	Hardness (N)	Adhesiveness (mJ)	Cohesiveness (-)	Gumminess (N)	Springiness (-)	Chewiness (N·mm)
T1	208.50 ± 5.50 ^bc^	20.11 ± 2.67 ^ab^	11.24 ± 1.27 ^ab^	30.66 ± 1.20 ^cd^	0.27 ± 0.01 ^b^	8.02 ± 0.21 ^cd^
T2	243.50 ± 6.50 ^ab^	19.39 ± 3.04 ^ab^	9.49 ± 3.31 ^bc^	46.04 ± 2.13 ^b^	0.33 ± 0.01 ^ab^	15.21 ± 0.94 ^b^
T3	219.50 ± 25.5 ^bc^	18.35 ± 4.78 ^ab^	8.75 ± 2.35 ^bc^	35.35 ± 5.94 ^c^	0.36 ± 0.02 ^a^	12.61 ± 1.61 ^bc^
T4	166.50 ± 4.50 ^d^	14.77 ± 2.51 ^b^	9.44 ± 1.17 ^bc^	20.71 ± 2.21 ^e^	0.27 ± 0.01 ^b^	5.64 ± 0.41 ^e^
TSF	191.50 ± 2.50 ^cd^	14.17 ± 0.95 ^b^	7.88 ± 1.02 ^c^	33.24 ± 1.33 ^cd^	0.29 ± 0.02 ^b^	9.52 ± 0.97 ^c^
TCF	276.00 ± 29.00 ^a^	22.17 ± 4.21 ^a^	13.59 ± 3.61 ^a^	54.84 ± 4.63 ^a^	0.35 ± 0.02 ^a^	18.85 ± 0.69 ^a^

T1–T4: semolina pasta; TSF: pasta with sorghum flour; TCF: pasta with cork oak flour. Values are expressed as mean ± standard deviation (n = 3). Different superscript letters within the same column indicate statistically significant differences (*p* < 0.05).

**Table 8:** A qualitative analysis of the phenolic compounds present in sorghum flour SF.

Formula	[M + H]^+^	[M + Na]^+^	[M − H]^−^	Fragments	Compounds	Rf (min)
C_10_H_8_O_4_	-	-	191.0379	176.0186; 148.0234 124.0461	Scopoletin	4.971
C_6_H_12_O_3_			131.0718	129.0567; 113.0564	Hydroxycaproic acid	5.209
C_9_H_10_O_3_	-	-	165.0559	147.0444; 119.0498; 103.0561	2-Hydroxy-3-phenylpropanoic acid (3-Phenyllactic acid)	8.225
C_16_H_12_O_6_	-	-	299.0597	165.0226; 133.0273	Luteolin methyl ether (7or5)	15.184
C_10_H_8_O_4_	-	-	191.0361	176.0124; 148.0210	Scopoletin isomer	16.255
C_15_H_12_O_6_	-	-	287.0606	151.0058; 135.0462 107.0156	Eriodictyol	18.969
C_15_H_10_O_6_	-	-	285.0415	151.0013; 133.0292; 107.0148	Luteolin	20.345
C_15_H_12_O_5_	-	-	271.0644	177.0259; 151.0031; 119.0519	Naringenin	22.301

**Table 9:** A qualitative analysis of the phenolic compounds present in cork oak flour CF.

Formula	[M + H]^+^	[M + Na]^+^	[M − H]^−^	Fragments	Compound	Rf [min]
C_7_H_12_O_6_			191.0563	173.0521; 127.0490	Quinic acid	1.905
C_7_H_6_O_5_			169.0145	125.0237	Gallic acid	2.334
C_6_H_8_O_7_			191.0207	111.0125	Citric acid	2.714
C_9_H_10_O_5_	-	-	197.0470	169.0125; 125.0228	Ethyl gallate	7.457
C_15_H_20_O_10_			335.0431	183.0329	Digallic acid methyl	12.530
C_8_H_8_O_5_			183.0228	168.0004; 124.0130	Methyl gallate	4.000
C_14_H_6_O_8_			301.0008	145.0276	Ellagic acid	14.502

**Table 10:** Content of polyphenols (TPC) per gallic acid and flavonoids (TFC) per rutin in the tested samples.

Sample	TPC (mg GAE/mL Extract)	TFC (mg QE/mL Extract)
S1	0.099 ± 0.004 ^g^	0.299 ± 0.013 ^f^
S2	0.123 ± 0.001 ^e^	0.328 ± 0.012 ^e^
S3	0.105 ± 0.002 ^g^	0.417 ± 0.027 ^d^
S4	0.118 ± 0.002 ^f^	0.581 ± 0.052 ^c^
SF	1.048 ± 0.056 ^b^	0.947 ± 0.031 ^b^
CF	1.715 ± 0.050 ^a^	1.615 ± 0.003 ^a^
T2	0.116 ± 0.002 ^f^	0.310 ± 0.010 ^f^
TSF	0.299 ± 0.015 ^d^	0.254 ± 0.011 ^g^
TCF	0.314 ± 0.015 ^c^	0.299 ± 0.000 ^f^

S1–S4—semolina; SF—sorghum flour; CF—cork oak flour; T2—pasta with semolina; TSF—pasta with sorghum flour; TCF—pasta with cork oak flour. Values are expressed as mean ± standard deviation (n = 3). Different superscript letters within the same column indicate statistically significant differences (*p* < 0.05).

**Table 11:** DPPH scavenging ability of raw materials and pasta by spectrophotometric method.

Sample	RSA After 10 Min (%)	RSA After 20 Min (%)	RSA After 30 Min (%)
S1	24.87 ± 1.12 ^g^	28.27 ± 0.53 ^g^	30.19 ± 0.08 ^g^
S2	27.53 ± 0.83 ^f^	31.68 ± 0.68 ^f^	34.40 ± 0.15 ^f^
S3	22.11 ± 1.00 ^h^	25.66 ± 1.08 ^h^	27.89 ± 1.16 ^h^
S4	31.44 ± 1.08 ^e^	36.90 ± 1.08 ^e^	40.17 ± 1.23 ^e^
SF	77.84 ± 0.50 ^b^	78.45 ± 0.50 ^b^	78.65 ± 0.50 ^b^
CF	91.10 ± 0.39 ^a^	91.32 ± 0.54 ^a^	91.43 ± 0.54 ^a^
T2	25.80 ± 0.75 ^g^	30.20 ± 0.60 ^f^	33.00 ± 0.20 ^f^
TSF	45.60 ± 0.29 ^d^	51.19 ± 0.44 ^d^	53.10 ± 0.49 ^d^
TCF	54.41 ± 0.64 ^c^	61.71 ± 0.93 ^c^	64.15 ± 0.93 ^c^

S1–S4—semolina; SF—sorghum flour; CF—cork oak flour; T2—pasta with semolina; TSF—pasta with sorghum flour; TCF—pasta with cork oak flour. Values are expressed as mean ± standard deviation (n = 3). Different superscript letters within the same column indicate statistically significant differences (*p* < 0.05).

**Table 12:** TLC-DPPH assay results. Activity of the analyzed extracts SF, TSF, CF, and TCF, expressed relative to the semolina S2 extract, with activity taken as 1.

Sample	After 0 Min	After 10 Min	After 30 Min	Reference	Description
SF	1.860 ^b^	1.867 ^b^	1.857 ^b^	1.000	Sorghum flour
TSF	1.362 ^c^	1.371 ^c^	1.372 ^c^	1.000	Pasta with sorghum flour
CF	2.789 ^a^	2.883 ^a^	2.887 ^a^	1.000	Cork oak flour
TCF	1.564 ^b^	1.566 ^b^	1.569 ^b^	1.000	Pasta with cork oak flour
S2	1.000 ^d^	1.000 ^d^	1.000 ^d^	1.000	Semolina
T2	1.090 ^cd^	1.115 ^cd^	1.112 ^cd^	1.000	Control pasta with semolina S2

SF—sorghum flour; TSF—pasta with sorghum flour; CF—cork oak flour; T2—pasta with semolina; TCF—pasta with cork oak flour; S2—semolina. Different superscript letters within the same column indicate statistically significant differences (*p* < 0.05).

## Data Availability

The original contributions presented in the study are included in the article/Supplementary Materials, further inquiries can be directed to the corresponding authors.

## Supplementary Materials

The following supporting information can be downloaded at: https://www.mdpi.com/article/10.3390/foods14162832/s1, Figure S1: TLC chromatogram of semolina extract (S2); Figure S2: TLC chromatogram of sorghum-enriched pasta extract (TSF); Figure S3: TLC chromatogram of cork oak-enriched pasta extract (TCF).
