# Supplementary material for: Physiochemical Characterization and Antioxidant Potential of Sorghum and Cork Oak as Valuable Additives to Traditional Trida Pasta
Source: Foods. 2025 Aug 15;14(16):2832. doi: 10.3390/foods14162832 (PMC12385945; doi:10.3390/foods14162832)

Supplementary Material

Physiochemical Characterization and Antioxidant Potential of Sorghum and Cork Oak as Valuable Additives to Traditional Trida Pasta

Rima Sabouni[1], Louiza Himed[2], Belkis Akachat[2], Agnieszka Wójtowicz[3*], Kamila Kasprzak-Drozd[4], Hacène Namoune[1] Salah Merniz[5], Maria D'Elia[6,7,8], Luca Rastrelli[6,7*], Anna Oniszczuk[4,]

Figure S1. TLC chromatogram of semolina extract (S2), processed with Sorbil TLC Videodensitometer V 2.3 software; scan performed after 30 minutes.

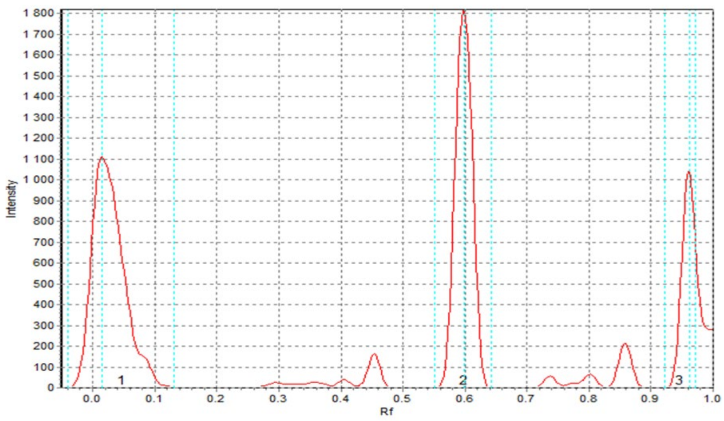

Figure S2. TLC chromatogram of sorghum-enriched pasta extract (TSF), processed with Sorbil TLC Videodensitometer V 2.3 software; scan performed after 30 minutes.

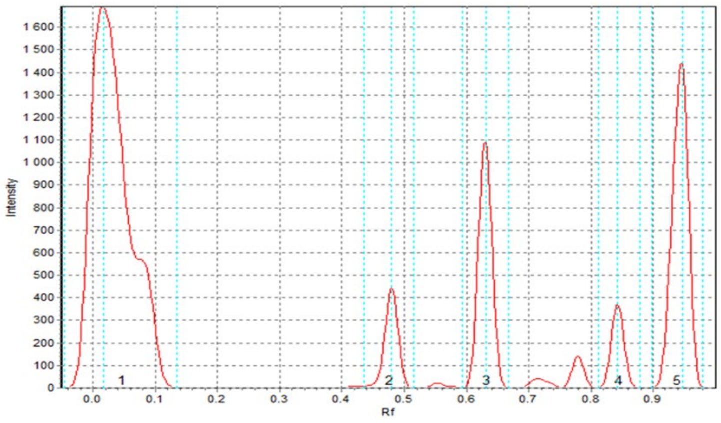

Figure S3. TLC chromatogram of cork oak-enriched pasta extract (TCF), processed with Sorbil TLC Videodensitometer V 2.3 software; scan performed after 30 minutes.

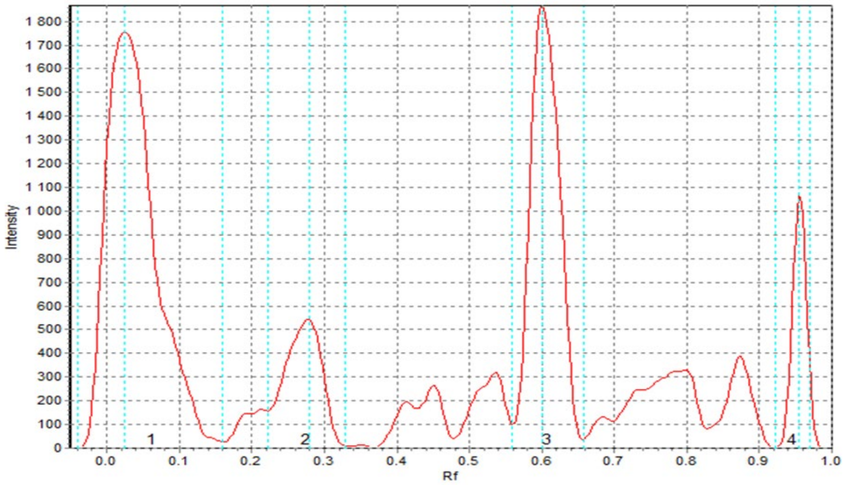

Supplement: Supplementary file 1 [file foods-14-02832-s001.zip › foods-3797980-supplementary.pdf]
